# Supplementary material for: Fine motor deficits in reading disability and language impairment: same or different?
Source: PeerJ. 2013 Nov 28;1:e217. doi: 10.7717/peerj.217 (PMC3845870; doi:10.7717/peerj.217)
Supplement: Supplemental Information 1 [file peerj-01-217-s001.doc]

##
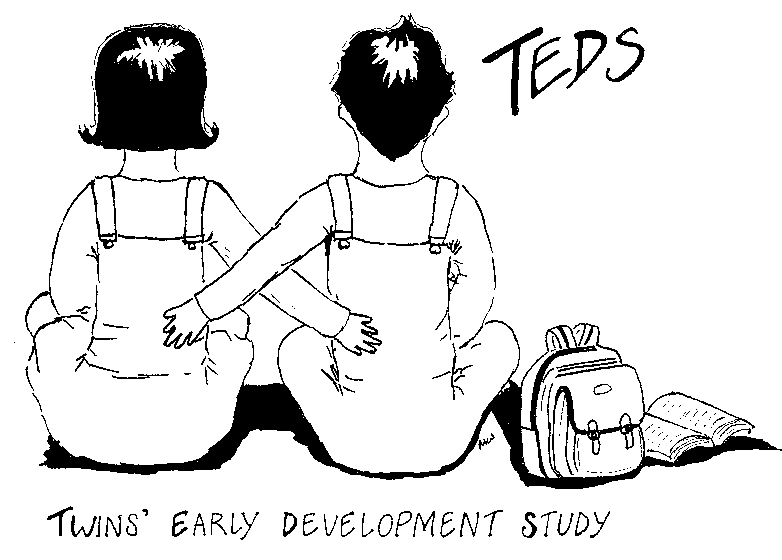


**TWINS EARLY DEVELOPMENT STUDY –follow-up at 9 to 10 years**

**CONSENT FORM**

Names of twins:

Date of birth:

Address:

Post Code:

Telephone: Mobile:

Children’s School(s):

Head Teacher:

Class Teacher(s):

School Address

Telephone Number:

## Please sign below

- I agree to take part in the 9 to 10-year-old follow-up of Twins Early Development Study, and for the children named above to be seen at home/school by members of the research team. YES / NO
- I understand that I can withdraw from the study at any time without having to give a reason.

Name (block capitals) of parent/guardian: ____________________________

Date: ___________ Signature: ________________________________

Please return in pre-paid envelope provided to: TEDS

Research Centre

113 Denmark Hill

FREEPOST LON 7567

London SE5 8YZ
