# Supplementary material for: Fine motor deficits in reading disability and language impairment: same or different?
Source: PeerJ. 2013 Nov 28;1:e217. doi: 10.7717/peerj.217 (PMC3845870; doi:10.7717/peerj.217)
Supplement: Table S1 [file peerj-01-217-s002.docx]

Supplemental Table S1.

SPSS script for mixed models analysis of dependent variable Purdue_raw, with factors LI and RD, and sex as covariate. Note that the analysis includes family_ID as a random factor: this is identical for two twins in a family. The analysis adjusts the degrees of freedom to take into account covariances between two members of a twin pair. For a detailed explanation of this approach see <http://davidakenny.net/dyad.htm>. In the terminology used by Kenny, twin is a “mixed variable” (see Topic 10).

MIXED Purdue_raw BY LI RD WITH sex

/CRITERIA=CIN(95) MXITER(100) MXSTEP(10) SCORING(1) SINGULAR(0.000000000001) HCONVERGE(0,

ABSOLUTE) LCONVERGE(0, ABSOLUTE) PCONVERGE(0.000001, ABSOLUTE)

/FIXED=LI RD LI*RD sex | SSTYPE(3)

/METHOD=REML

/PRINT= SOLUTION

/RANDOM=INTERCEPT | SUBJECT(family_ID) COVTYPE(VC)

/EMMEANS=TABLES(LI)

/EMMEANS=TABLES(RD) .
